# Tooth brushing for a longer and healthier life

**DOI:** 10.1007/s12471-016-0802-1

**Published:** 2016-02-08

**Authors:** E. E. van der Wall

**Affiliations:** Netherlands Society of Cardiology/Holland Heart House, Moreelsepark 1, 3511 EP Utrecht, The Netherlands

Patients with coronary artery disease (CAD) who have no teeth have nearly double the risk of death compared with CAD patients with all of their teeth, according to research performed at the Uppsala Clinical Research Center, Uppsala, Sweden, and published in the European Journal of Preventive Cardiology in December 2015 [1].

The study of 15,456 patients from 39 countries found that levels of tooth loss were linearly associated with increasing rates of death and stroke. In recent decades, periodontal disease has emerged as a potential risk factor for CAD. Already in 1993, De Stefano et al. showed that dental disease was associated with an increased risk of CAD [2]. Similar to CAD, periodontal disease is a widespread disease with chronic inflammatory properties, influenced by numerous risk factors, of which lifestyle, poor oral health habits, age, smoking, diabetes and socioeconomic status are the most significant ones [3–10]. In patients with type 1 diabetes, periodontal disease is an independent predictor of long-term progression of calcium deposits in the coronary arteries [11]. Recently, genetic evidence for plasminogen as a shared genetic risk factor of CAD and periodontitis was shown [12]. Periodontal disease is the main cause of tooth loss and is related to poor dental hygiene, being one of the strongest risk factors for the occurrence of periodontal disease.

The study by Vedin et al. [1] is the first study to prospectively assess the relationship between tooth loss and outcomes in patients with CAD. The results are derived from a substudy of the STABILITY trial [13, 14], which evaluated the effects of the lipoprotein-associated phospholipase A2 (Lp-PLA2) inhibitor darapladib versus placebo in patients with CAD. At the start of the study all patients completed a questionnaire about lifestyle factors, psychosocial factors, and their number of teeth in five different categories: (26–32 i.e. considered to have all teeth, 20–25, 15–19, 1–14 teeth and no teeth). Patients were followed for an average of 3.7 years. Associations between tooth loss and outcomes were calculated after adjusting for cardiovascular risk factors and socioeconomic status. The primary outcome was major cardiovascular events being a composite of cardiovascular death, myocardial infarction and stroke.

Patients who lost their teeth were older, smokers, female, less active and more likely to have diabetes, high blood pressure, more body fat and a lower level of education. During follow-up there were 1543 major cardiovascular events, 705 cardiovascular deaths, 1120 deaths from any cause and 301 strokes. After adjusting for cardiovascular risk factors and socioeconomic status, every increase in category of tooth loss was associated with a 6 % increased risk of major cardiovascular events, 17 % increased risk of cardiovascular death, 16 % increased risk of all-cause death and 14 % increased risk of stroke.

After adjusting for risk factors and socioeconomic status, the group with no teeth had a 27 % increased risk of major cardiovascular events, 85 % increased risk of cardiovascular death, 81 % increased risk of all-cause death and 67 % increased risk of stroke compared with those with all of their teeth.

The authors commented that the risk increase was linear with the highest risk in those with no remaining teeth. For example, the risks of cardiovascular death and all-cause death were almost double to those in patients with all teeth remaining. Coronary heart disease and periodontal disease share many risk factors such as smoking, unhealthy lifestyle and diabetes but after adjusting for these factors an apparently independent relationship between the two conditions was found. Many patients in the study had lost teeth: around 16 % of patients had no teeth and about 40 % were missing half of their teeth. Several mechanisms by which periodontal disease may impact CAD and cerebral strokes have been proposed, including detrimental effects on endothelial function, atherosclerosis progression and plaque stability [15, 16]. Systemic inflammation generated by periodontal disease has been suggested as a possible causative pathway based on observations of increased levels of inflammatory markers in patients with periodontal disease [17]. The inflammation from periodontal disease is thought to trigger the atherosclerotic process and may explain the associations observed in the present study. However, the associations between periodontal disease and CAD included several cardiovascular outcomes and were independent of multiple risk factors. Therefore, the possibility of residual confounding or influence by unmeasured causal factors cannot be excluded.

During the study period 746 patients experienced a myocardial infarction. There was a numerically increased risk of myocardial infarctions for every increase in tooth loss. However, this finding was not significant after adjustment for risk factors and socioeconomic status. The occurrence of this phenomenon was not fully clear to the authors as they found strong associations with other cardiovascular outcomes including stroke.

To summarise, in this large global cohort of patients with CAD, self-reported tooth loss predicted adverse cardiovascular outcomes and all-cause death independent of cardiovascular risk factors and socioeconomic status [1]. Every increased level of tooth loss was associated with a 6 % increased risk of major cardiovascular events, and an approximately 15 % higher risk of cardiovascular death, death from any cause and stroke. Overall, CAD patients with no teeth had nearly twice the risk of death to those patients with all of their teeth. The relationship between tooth loss and outcomes in CAD patients has not previously been evaluated in a prospective study. However, it is too premature to conclude that periodontal disease directly causes adverse events in CAD patients. According to the authors, tooth loss remains an easy and inexpensive way to identify patients at higher risk who need more intense dental instructions as poor dental hygiene remains one of the strongest risk factors for periodontal disease. As a result, one should intensively hang onto tooth brushing and flossing in order to live longer and healthier!
